# Effects of an L-Leucine-Rich Diet on Liver and Kidneys in a Doxorubicin Toxicity Model

**DOI:** 10.3390/life13091823

**Published:** 2023-08-29

**Authors:** Poliana Rodrigues Alves Duarte, Rodrigo Rodrigues Franco, Danielle Diniz Vilela, Douglas Carvalho Caixeta, Adriele Vieira de Souza, Simone Ramos Deconte, Clesnan Mendes-Rodrigues, Thiago Montes Fidale, Foued Salmen Espindola, Renata Roland Teixeira, Elmiro Santos Resende

**Affiliations:** 1 Faculdade de Medicina, Universidade Federal de Uberlândia, Uberlândia 38400-902, MG, Brazil; poliana.duarte@ufcat.edu.br (P.R.A.D.); simonerd@ufu.br (S.R.D.); clesnan@ufu.br (C.M.-R.); thiagofidale@ufcat.edu.br (T.M.F.); 2 Departamento de Medicina, Universidade Federal de Catalão, Catalão 75706-881, GO, Brazil; rodrigo_franco@ufcat.edu.br; 3 Instituto de Biotecnologia, Universidade Federal de Uberlândia, Uberlândia 38400-902, MG, Brazil; danielledvilela@ufu.br (D.D.V.); douglas.caixeta@ufu.br (D.C.C.); adrielevieira@ufu.br (A.V.d.S.)

**Keywords:** doxorubicin, L-leucine, antioxidant status, BCAA, histological parameters

## Abstract

Supplements and diets containing L-leucine, a branched-chain amino acid, have been considered beneficial for controlling oxidative stress and maintaining cardiac tissue in toxicity models using doxorubicin, a drug widely used in cancer treatment. However, there is a lack of studies in the literature that assess the effects of this diet on other organs and tissues, such as the liver and kidneys. Therefore, this study aimed to evaluate the effects of a leucine-rich diet on the liver and kidneys of healthy rats submitted to the doxorubicin toxicity model by analyzing biomarkers of oxidative stress and histological parameters. The animals were divided into four groups: naive, doxorubicin, L-leucine, and doxorubicin + L-leucine, and the diet was standardized with 5% L-leucine and a dose of 7.5 mg/kg of doxorubicin. We evaluated tissue injury parameters and biomarkers of oxidative stress, including enzymes, antioxidant profile, and oxidized molecules, in the liver and kidneys. Although some studies have indicated benefits of a diet rich in L-leucine for the muscle tissue of animals that received doxorubicin, our results showed that the liver was the most affected organ by the L-leucine-rich diet since the diet reduced its antioxidant defenses and increased the deposit of collagen and fat in the hepatic tissue. In the kidneys, the main alteration was the reduction in the number of glomeruli. These results contribute to the scientific literature and encourage further studies to evaluate the effects of an L-leucine-rich diet or its supplementation, alone or combined with doxorubicin using an animal model of cancer. Therefore, our study concludes that the leucine-rich diet itself was harmful and, when co-administered with doxorubicin, was not able to maintain the antioxidant defenses and tissue structure of the evaluated organs.

## 1. Introduction

Cancer is the world’s second leading cause of death, accounting for approximately 10 million deaths in 2020 [1]. This disease can occur in many tissues and organs [2], and cancer cells result from the interaction between an organism’s genetic factors and physical, chemical, and biological carcinogens [3]. These abnormal cells can grow uncontrollably, increasing their number and invading adjacent parts of the body and/or spread to other organs (metastasizing) [4]. 

Many studies have been and continue to be carried out in search of new therapies against cancer. However, chemotherapy remains one of the most used treatments to date [5]. One of the most commonly used chemotherapeutics is the antibiotic doxorubicin, which belongs to the anthracycline group and was isolated from the bacteria *Streptomyces peucetius* var. caesius in 1970 [6]. 

Ref. [7] describe that doxorubicin has multiple mechanisms of action, including the generation of free radicals, which promote oxidative damage to the membranes and proteins of cancer cells. After acting, this drug can be metabolized in the liver and then excreted in the bile and urine [8]. Despite its common use, doxorubicin not only has toxic effects on cancers cells but also various healthy tissues and organs, especially the liver, kidneys, and heart [9]. 

Therefore, the drug is commonly used in toxicity models, as it can alter biochemical [10] and morphological markers [9]. In this context, many researchers have sought therapies to alleviate the toxic effects of doxorubicin with emphasis on co-treatment with supplements and diets based on the branched-chain amino acids (BCAA) L-leucine, L-isoleucine, and L-valine [11,12]. 

L-leucine is the most abundant BCAA found in animals and has great importance for tissues [13], as it can stimulate protein synthesis and prevent muscle atrophy caused by doxorubicin [14] and cancer [15]. Although these data indicate the benefits of an L-leucine-rich diet for the muscle tissue of animals that received doxorubicin, it is noted that the literature needs more conclusive studies to evaluate the biological effects of L-leucine and its co-treatment with doxorubicin on the liver and kidneys.

However, ref [16] indicate that BCAA excess is associated with non-alcoholic fatty liver disease and oxidative injury. In addition, clinical studies suggest that BCAA excess may harm liver structure and function [17,18]. Furthermore, the study of [19] indicates that BCAA excess rapidly interfered with renal function, decreasing the glomerular number and filtration rate. 

On another hand, Zhenyukh et al. (2018) [20] suggest that L-leucine and other BCAA activation pathways stimulate reactive oxygen species (ROS) and production of pro-inflammatory factors. The liver is essential to metabolize, through oxidations and other chemical reactions, the residues of cellular metabolism, drugs, and their intermediates; the kidneys are also important for the excretion of these components [8].

So, hypothetically, a diet rich in L-leucine, alone or when co-treated with doxorubicin, may have degenerative effects on the liver and kidneys, despite the general benefits of this amino acid for the heart and muscles, demonstrating the importance of studies that evaluate its systemic effects and not just local ones.

Therefore, the main objective of this study was to evaluate the effects of an L-leucine-rich diet on the liver and kidneys of healthy rats using the doxorubicin toxicity model. The study aimed to evaluate biomarkers of oxidative stress and histological parameters to assess the extent of damage caused.

## 2. Material and Methods

### 2.1. Animals and Groups 

Thirty-six randomly selected male Wistar rats, approximately 12 weeks old and weighing between 250 g and 300 g were used. The animals were housed in the institution’s vivarium, under a controlled environment (temperature, humidity, and light–dark cycle), with free access to food and water. They were monitored daily, and the protocol was approved by the Ethics and Animal Utilization Committee of the Federal University of Uberlândia, according to protocol 115/14. The animals were randomly divided into four groups, based on [11]: NAIVE, eight animals; DOX (doxorubicin), ten animals; LEU (L-leucine), eight animals; and DOX + LEU (doxorubicin + L-leucine), ten animals. The LEU and DOX + LEU groups had their daily leucine concentration raised to 5%.

### 2.2. L-Leucine-Rich Diet and Doxorubicin Treatment

The diets were prepared in the laboratory with control for risks of chemical and biological contamination. The standard diet was formulated following the recommendations of the American Institute of Nutrition [21] and adapted to the rodent’s growth, gestation, and lactation. The casein used had an adequate protein concentration (85.07%), according to the supplier’s report. The diet had a minimum L-leucine concentration of 1.5 g/100 g (1.5%) as described by Reeves et al. in 1993 and was used for NAIVE and DOX groups. However, for the L-leucine-rich diet, administrated to L-leucine-treated groups (LEU and DOX + LEU), the standard diet included an additional 5.0 g/100 g (5%) of L-leucine [11]. 

The dietary nutrients were provided as already described in a previous experiment [11]. The diets were isocaloric (395.0 kcal/100 g), containing similar values of macronutrients in the standard diet (protein: 20%, carbohydrate: 64%, and fat: 16%) and in the L-leucine-rich diet (protein: 25%, carbohydrate: 59%, and fat: 16%). The animals of the naïve and LEU groups received their respective diets and water ad libitum for 42 days, and the animals of the DOX and DOX + LEU groups received their respective diets 14 days before, during, and after starting doxorubicin applications. 

The DOX and DOX + LEU groups received intraperitoneal injections of doxorubicin hydrochloride (FaulDoxo, Libbs Farmacêutica, Embú, São Paulo, Brazil) three times a week, for two weeks, reaching a cumulative dose of 7.5 mg/kg adapted from [22]. The NAIVE and LEU groups received a 0.9% saline solution in the same period as the doxorubicin-treated animals (DOX and DOX + LEU). 

### 2.3. Tissue Processing 

After 42 days of experiment, the animals were anesthetized using Ketamine 74 mg/kg and Xylazine 8 mg/kg by intraperitoneal injection. Euthanasia was confirmed after total blood collection by puncture of the abdominal aorta. The blood samples were submitted to liver and kidney function tests (aspartate aminotransferase, alanine aminotransferase, urea, and creatinine); however, the data did not show significant differences between the groups (Table S1 in supplementary materials). 

The liver and kidneys were removed through an abdominal incision and promptly processed to avoid the degradation of enzymes or oxidation of biomolecules in these tissues. The liver was divided into two parts, the no hepatic square lobe part being frozen until the performance of the oxidative stress tests and the other part, the hepatic square lobe, was fixed in formalin solution (4%) (pH 7.2) for two hours for histological analysis. The right kidney was frozen until the performance of the oxidative stress tests, and the left kidney was fixed in formalin solution (4%) (pH 7.2) for two hours for histological analysis. 

### 2.4. Biomarkers of Oxidative Stress

The organs were homogenized separately in a 20 mM sodium phosphate buffer (pH 7.4) containing 140 mM KCl in the proportion (1:10 g/mL). The homogenates were centrifuged at 800× *g* at 4 °C for 10 min, and the supernatant was collected for analysis of oxidative stress biomarkers. The Bradford method (Bradford, 1976), with an analytical curve made with bovine serum albumin, was used to determine the total protein concentration in the homogenates. Absorbance was measured at 570 nm in a 96-well plate reader spectrophotometer [23].

#### 2.4.1. Catalase 

Catalase (CAT) catalysis was evaluated by measuring the decomposition of hydrogen peroxide (H_2_O_2_). Samples were incubated with a Triton X-100 solution (10%) and 10 mM potassium phosphate buffer (pH 7.0) containing H_2_O_2_ (0.2%). The decay of H_2_O_2_ concentration was measured at 240 nm for 10 min in a 96-well plate-reading spectrofluorometer [23]. 

#### 2.4.2. Superoxide Dismutase 

Superoxide dismutase (SOD) catalysis was evaluated by measuring the inhibition of pyrogallol autoxidation. In a basic medium, pyrogallol undergoes partial oxidation and releases the superoxide ion. The superoxide ion may completely oxidize pyrogallol after it is formed, leading to the formation of a colored product identified at 420 nm. SOD is capable of converting the superoxide ion into hydrogen peroxide, preventing the complete oxidation of pyrogallol. Samples were incubated with a 50 mM Tris-HCl buffer (pH 8.2) containing 1 mM of EDTA, catalase (80 U/mL), and 24 mM of pyrogallol. The kinetic reading was evaluated for 10 min and read in a 96-well plate reader spectrophotometer at 420 nm. An analytical curve made with SOD was used [24].

#### 2.4.3. Glutathione Peroxidase 

Glutathione peroxidase (GPx) catalysis was quantified considering the consumption of NADPH during the conversion of oxidized glutathione to reduced glutathione. Samples were mixed with a GPx buffer solution containing 100 mM of potassium phosphate (pH 7.7) and 1 mM of EDTA. In addition, 40 mM of sodium azide, 100 mM of GSH diluted in metaphosphoric acid solution (5%), 4.5 U of glutathione reductase diluted in GPx buffer, 2 mM of NADPH diluted in sodium bicarbonate solution (5%), and 0.5 mM of tert-butyl were added to the mixture. NADPH consumption was read using a 96-well plate reader spectrofluorometer for 10 min at 340 nm [23]. 

#### 2.4.4. Glutathione Reductase 

Glutathione reductase (GR) catalysis was measured considering the consumption of the NADPH present in the samples, which were diluted in GR buffer (200 mM of sodium phosphate pH 7.5 and 6.3 mM of EDTA) and 2 mM of NADPH. The kinetic assay was read in a 96-well plate-reading spectrofluorometer at 340 nm for 10 min [24].

#### 2.4.5. Reduced Glutathione 

The samples were initially treated with a metaphosphoric acid solution, to precipitate proteins from the homogenate, and then centrifuged at 7000× *g* for 10 min. A methanolic solution of ortho-phthaldialdehyde (1 mg/mL) and 100 mM sodium phosphate buffer (pH 8.0) with 5 mM of EDTA was added to the supernatant. The assay was evaluated in a 96-well plate-reading spectrofluorometer at 350 nm (excitation) and 420 nm (emission). The analytical curve was constructed with reduced glutathione (GSH) as a standard [25]. 

#### 2.4.6. Glucose-6-Phosphate Dehydrogenase

Glucose-6-phosphate dehydrogenase (G6PDH) catalysis was evaluated by measuring NADPH synthesis, quantifiable by the increase in absorbance at 340 nm. Samples were mixed with a 100 mM Tris-HCl buffer solution (pH 7.5) containing 0.5 mM of NADP+, magnesium chloride, and 1 mM of glucose-6-phosphate. The kinetic assay was read for 10 min in a 96-well plate reader spectrophotometer [25].

#### 2.4.7. Lipid Peroxidation

The samples were mixed with solutions of thiobarbituric acid (0.67%) and trichloroacetic acid (10%) and incubated for 120 min in a water bath. After this period, the organic phase of the samples, removed using n-butanol, was read at 515 nm (excitation) and 553 nm (emission) using a 96-well plate reader spectrofluorometer. The level of lipid peroxidation (TBARS) was obtained by comparing the results with a standard curve made with malondialdehyde [26].

#### 2.4.8. Thiol Groups

Samples were incubated for 30 min with a 1 mM phosphate buffer solution (pH 7.4) and 10 mM dithionitrobenzoic acid (DTNB) solution, diluted in a 0.2 M potassium phosphate buffer solution (pH 8.0). The total content of thiol groups was measured in a 96-well plate reader spectrophotometer at 412 nm [27]. 

### 2.5. Histological Analysis 

The fixed samples were dehydrated in increasing concentrations of ethanol (70, 80, 90, and 100%) followed by two changes of xylol, and then embedded in paraffin to form blocks. The histological sections were cut into 5 μm slides and mounted on glass slides. Before staining, the slices were dewaxed in two xylol exchanges, hydrated in decreasing concentrations of alcohol (100, 90, 80, and 70%) and washed in running water. To visualize the general morphology, quantification of collagen, and fat deposition, the sections were stained with a Gomori trichrome, using a red picrosirius color. The sections were visualized, and the images were obtained using a 40× objective (liver) and 10× objective (kidney), through the Leica Microsystems Inc., Wetzlar, Germany camera attached to the microscope [24].

### 2.6. Statistical Analysis

The statistical analysis was performed using GraphPad Prism software, version 8.0.2. The results were found as mean ± standard error of the mean, and the normality of the data was tested using the Shapiro–Wilk test. These were compared using the analysis of variance (ANOVA). Tukey’s test was used to determine differences between groups. Differences were considered significant when *p* < 0.05. Adjusted *p* Value, for each method, is shown in Tables S2–S10 in supplementary materials.

## 3. Results

### 3.1. L-Leucine-Rich Diet Can Reduce the Antioxidant Defenses Activities 

**Figure 1:**
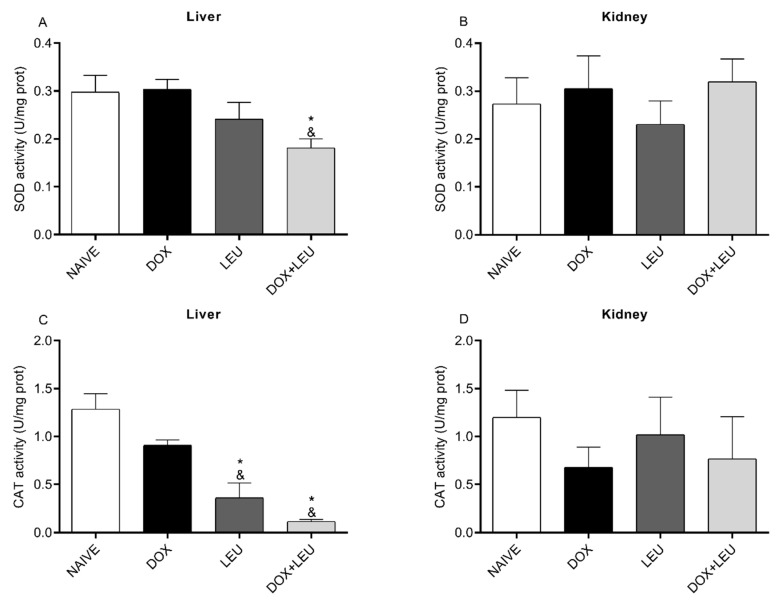
Superoxide dismutase (**A**,**B**) and catalase (**C**,**D**) activities in the liver and kidney of control rats (NAIVE), supplemented with doxorubicin (DOX), leucine (LEU) and doxorubicin + leucine (DOX + LEU). ANOVA followed by Tukey’s test. Values expressed as mean ± standard error of the mean. * vs. NAÏVE; & vs. DOX. The differences were considered significant when *p* < 0.05.

As shown in Figure 1A, SOD activity decreased in the liver of the DOX + LEU compared to the DOX and NAIVE groups. The pattern of CAT activity in the liver decreased in LEU and DOX + LEU groups compared with DOX and NAIVE groups (Figure 1C). No difference was observed in the kidney of the analyzed groups (Figure 1B,D). 

**Figure 2:**
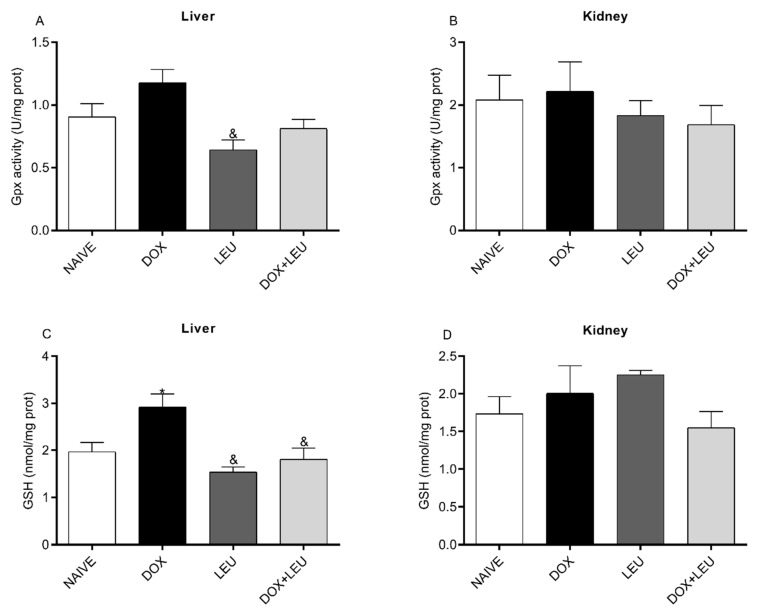
The activity of glutathione peroxidase (GPx) (**A**,**B**) and levels of reduced glutathione (GSH) (**C**,**D**) in the liver and kidney of control rats (Naive), supplemented with Doxorubicin (DOX), Leucine (LEU) and Doxorubicin + Leucine (DOX + LEU). ANOVA followed by Tukey’s test. Values expressed as mean ± standard error of the mean. * vs. NAIVE; & vs. DOX. The differences were considered significant when *p* < 0.05.

As shown in Figure 2A, GPx activity decreased in the liver of the LEU group compared to the DOX group. In addition, the GSH levels in the liver decreased in NAIVE, LEU, and DOX + LEU compared with the DOX group (Figure 2C). No difference was observed in the kidney of the analyzed groups (Figure 2B,D). 

**Figure 3:**
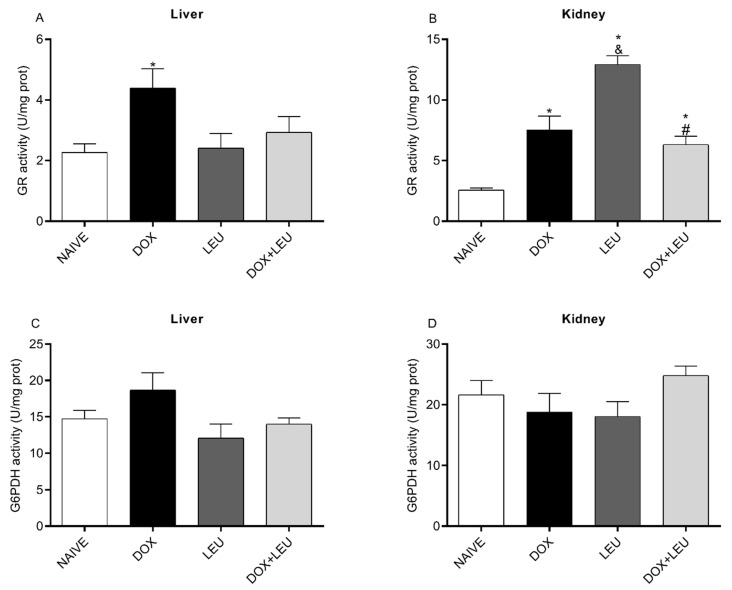
The activity of glutathione reductase (GR) (**A**,**B**) and glucose-6-phosphate dehydrogenase (G6PDH) (**C**,**D**) in the liver and kidney of control rats (NAIVE), supplemented with doxorubicin (DOX), leucine (LEU) and doxorubicin + leucine (DOX + LEU). ANOVA followed by Tukey’s test. Values expressed as mean ± standard error of the mean. * vs. NAÏVE; # vs. L; & vs. DOX. The differences were considered significant when *p* < 0.05.

As shown in Figure 3A, GR activity decreased in the liver of the NAIVE group compared to the DOX group. On the other hand, GR activity increased in DOX, LEU, and DOX + LEU groups compared to the NAIVE group, increased in LEU group compared to the DOX group, and decreased in DOX + LEU group compared to the LEU group in the kidney (Figure 3B). No difference was observed in G6PDH activity in the liver or kidney of the analyzed groups (Figure 3C,D). 

**Figure 4:**
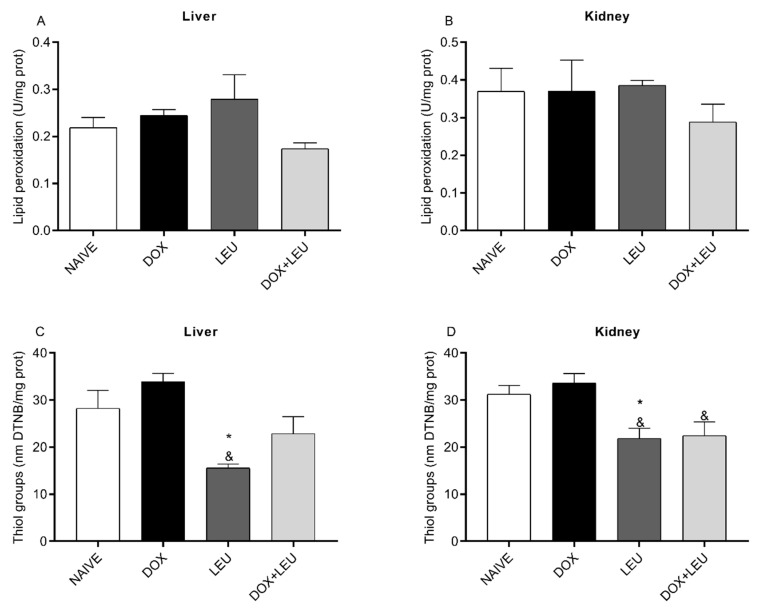
Levels of lipid peroxidation (**A**,**B**) and thiol groups (**C**,**D**) in the liver and kidney of control rats (NAIVE), supplemented with doxorubicin (DOX), leucine (LEU) and doxorubicin + leucine (DOX + LEU). ANOVA followed by Tukey’s test. Values expressed as mean ± SEM. * vs. C; & vs. D. The differences were considered significant when *p* < 0.05.

No statistical difference was observed in lipid peroxidation levels in the liver or kidney of the analyzed groups (Figure 4A,B). The content of thiol groups in the liver decreased in the LEU group compared to the NAIVE and DOX groups (Figure 4C). In the kidney, the content of thiol groups decreased in the DOX + LEU group compared to DOX group and decreased in the LEU group compared to the NAIVE and DOX groups (Figure 4D). 

### 3.2. L-Leucine-Rich Diet May Alter Liver and Kidney Tissue Structure

**Figure 5:**
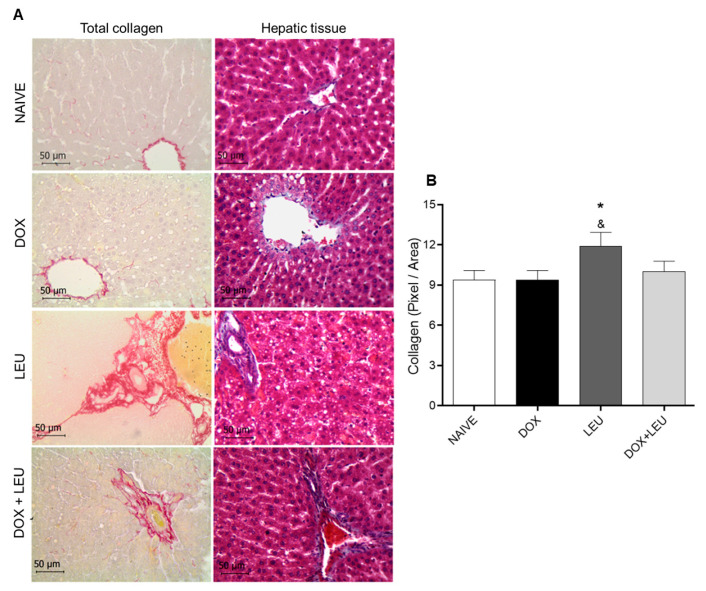
(**A**) Hepatic sections stained with picrosirius red (total collagen) and sections stained with Gomori’s trichrome (40×) (Hepatic tissue). (**B**) Total collagen in pixel/area. ANOVA followed by Tukey’s test. Values expressed as mean ± standard error of the mean. * vs. NAÏVE; & vs. DOX. The differences were considered significant when *p* < 0.05.

As shown in Figure 5A, it is possible to observe a triacylglycerol accumulation in the liver in the LEU and DOX + LEU groups. Figure 5A,B show the collagen deposition that occurred in the liver of the LEU group compared to NAIVE and DOX groups.

**Figure 6:**
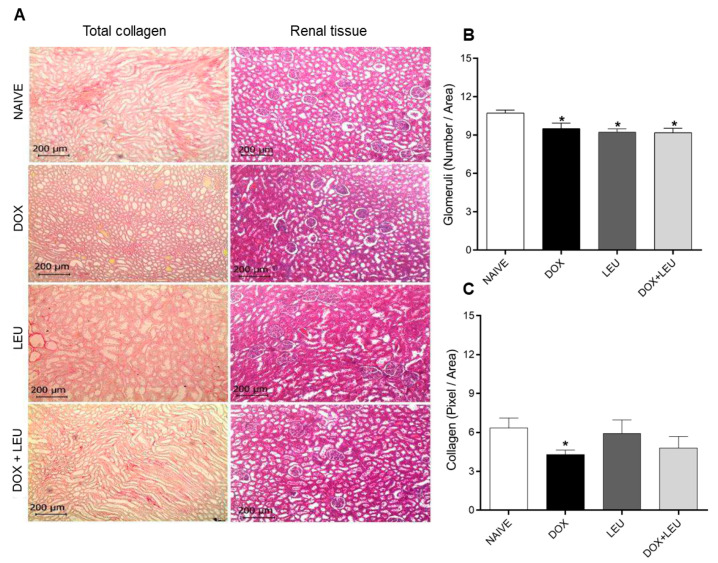
(**A**) Kidney slides stained with picrosirius red (total collagen) and slides stained with Gomori’s trichrome (renal tissue) (10×). (**B**) The number of glomeruli/area and (**C**) the total collagen in pixel/area. ANOVA followed by Tukey’s test. Values expressed as mean ± standard error of the mean. * vs. NAIVE. The differences were considered significant when *p* < 0.05.

As for the renal tissue, degenerations were not observed as shown in Figure 6A, but Figure 6B shows a reduction in the number of glomeruli numbers in the DOX, LEU, and DOX + LEU groups compared to the NAIVE group. There were less collagen deposits in the kidneys of the DOX group when compared to the NAIVE group.

## 4. Discussion

In this study, we evaluated the biomarkers of oxidative stress and histological parameters in the liver and kidneys of healthy rats treated with an L-leucine-rich diet, with 5% of L-leucine, using the chemotherapy drug doxorubicin at a dose of 7.5 mg/kg in a toxicological model. The literature contains studies that describe the positive effects of using supplements based on leucine and other BCAAs, to avoid protein catabolism and, consequently, maintain muscle fibers and strength in various health conditions and diseases, including cancer [15,28]. According to ref [15], an L-leucine-rich diet can reduce mitochondrial dysfunction, preserve skeletal muscle morphology, and protect against cachexia’s effects in a cancer model. 

Other studies suggest that L-leucine supplementation protects cardiomyocytes against degeneration promoted by doxorubicin. According to [11], an L-leucine-rich diet attenuates the heart failure caused by doxorubicin and promotes the maintenance of interstitial collagen fibers. According to [14], a formulation containing L-leucine was able to prevent oxidative damage and mitochondrial dysfunction generated by doxorubicin in cardiac tissue and cardiomyocytes. 

The safety and feasibility of using supplements depend on many important variables such as the individual’s age, lifestyle, and health [29,30]. Moreover, the use of medications that may interact with the supplements should also be considered [31]. Recent studies indicate that the amino acid L-leucine may be essential for the metabolism of cancer cells, contributing to the progression of cancer [32,33,34]. However, some studies suggest that L-leucine treated with doxorubicin may have beneficial effects in individuals with cancer [35,36]. 

Discussions are necessary on the systemic effects of supplementation since the compounds have different pharmacokinetic and pharmacodynamic mechanisms depending on the organ [18,37]. This study emphasizes the importance of further information on this topic, demonstrating that the L-leucine-rich diet may have contributed to altering the activity of biomarkers of oxidative stress and the analyzed histological parameters, with the liver being the most affected organ. 

The initial production of free radicals occurs with the formation of superoxide ions (O_2_^•−^), which can be reduced to H_2_O_2_ with SOD [38]. H_2_O_2_ can be converted into water (H_2_O) and oxygen gas (O_2_) with catalase or into two molecules of H_2_O with the glutathione system [39]. This study indicates that an L-leucine-rich diet reduces CAT activity and, when administered with doxorubicin, reduces SOD and CAT in the liver. According to [40], L- leucine is an amino acid capable of activating the rapamycin complex 1 (mTORC1), responsible for phosphorylating and inactivating the SOD1 (CuZnSOD) [41]. 

Doxorubicin is capable of inhibiting the expression of SOD and CAT genes and reducing the production of these antioxidant enzymes [42]. Moreover, doxorubicin can stimulate O_2_^•−^ production with mitochondrial NADPH oxidase [43]. The low amount and activity of the SOD allow the O_2_^•−^ accumulation, a radical that blocks the CAT activity [44]. 

The glutathione system is also able to reduce the H_2_O_2_ produced by SOD, requiring GSH, GPx, and GR [45]. The G6PDH enzyme, present in the pentose pathway, produces NADPH—which is necessary for the production of GSH—in a reaction catalyzed by GR [46]. The GSH molecule will provide electrons to reduce H_2_O_2_ into two H_2_O molecules, in a reaction catalyzed by GPx [45]. Our results indicate that an L-leucine-rich diet, when administered together with doxorubicin, reduces GSH levels. However, when administered alone, it reduces GSH levels and GPx activity. This may occur due to the reduction in the concentration of NADPH available to the glutathione system. 

The scientific literature indicates that doxorubicin stimulates the activity of mitochondrial NADPH oxidase, which consumes NADPH molecules [43]. In addition, the final metabolism of L-leucine in the liver can generate a carbon skeleton useful for triacylglycerol and cholesterol biosynthesis [47], including reactions that consume NADPH [48]. Interestingly, no treatment altered the activity of the G6PDH enzyme, but the group treated with doxorubicin increased GR activity in the liver and reduced in the kidneys. Furthermore, GR was also increased in the kidneys of the group treated only with L-leucine.

If the amount of these radicals exceeds the capacity of the described antioxidant mechanisms, proteins and lipids can be oxidized, reducing the number of sulfhydryl groups and increasing the amount of malondialdehyde, respectively [49,50]. The lipid peroxidation analyses did not show statistical differences between the groups but suggest that the L-leucine-rich diet alone tends to increase the levels of malondialdehyde in the liver. 

The concentration of sulfhydryl groups was reduced in the liver of the same group, possibly because they protect the organ against lipid peroxidation [51]. Furthermore, the L-leucine-rich diet and its co-treatment with doxorubicin reduced the levels of sulfhydryl groups in the kidneys but did not increase the lipid peroxidation content. Ref [20] suggest that L-leucine and other BCAAs promote endothelial dysfunction through increased reactive oxygen species generation and possibly consume sulfhydryl groups in glomeruli. 

The reduction in the cell’s antioxidant status indicates that there are more free radicals than the cell can fight, and with that, the free radicals can cause tissue damage, such as changes in collagen content or tissue necrosis [52]. The L-leucine-rich diet increased the production of collagen in the liver of the analyzed animals but did not trigger alterations in the kidneys. Conversely, the group co-treated with doxorubicin showed a tendency to reduce the production of collagen in the liver, but only the doxorubicin group showed a reduction in the production of this protein in the kidneys. 

L-leucine stimulates mTORC1, which in turn promotes collagen production [53], explaining the results. According to [54], doxorubicin reduced collagen production and the number of heart fibroblasts. In addition, initial studies with doxorubicin indicated that the molecule could inhibit the action of the prolyl 4-hydroxylase enzyme, responsible for collagen proline hydroxylation [55,56]. 

We also observed the accumulation of fat in the liver of animals that received an L-leucine-rich diet and with or without doxorubicin. Excessive leucine can contribute to hepatic lipogenesis, and the accumulation of fat in the liver may be favored by doxorubicin, which has been shown to inhibit beta-oxidation [10]. The histological analysis also showed a reduction in the number of glomeruli in all groups, except in the naive group.

Ref. [19] reported that excess branched-chain amino acids (BCAAs), including leucine, rapidly interfere with renal function, decreasing the glomerular filtration rate and promoting renal fibrosis. Conversely, ref. [57] suggest that doxorubicin-induced alterations in kidney function decrease the number of glomeruli and renal filtration. These results contribute to the current understanding of the systemic effects of an L-leucine-rich diet containing 5% leucine, providing new information about the biological effects on the liver and kidneys in a model of toxicity using the chemotherapeutic drug doxorubicin.

## 5. Conclusions

Our results suggest that an L-leucine-rich diet mainly affects the antioxidant defenses and tissue structure of the liver in healthy animals treated with doxorubicin. This information raises new discussions about the systemic effects of supplementation and opens new perspectives for future studies that can evaluate the potential benefits of co-treatment with L-leucine and doxorubicin in an animal model of cancer.

## Data Availability

Data may be available upon request.

## Supplementary Materials

The following supporting information can be downloaded at: https://www.mdpi.com/article/10.3390/life13091823/s1, Table S1: Liver and kidney function biomarkers; Table S2: Adjusted P Value for superoxide dismutase (SOD) test in liver and kidney; Table S3: Adjusted P Value for catalase (CAT) test in liver and kidney; Table S4: Adjusted P Value for glutathione peroxidase (GPx) test in liver and kidney; Table S5: Adjusted P Value for reduced glutathione (GSH) test in liver and kidney; Table S6: Adjusted P Value for glutathione reductase (GR) test in liver and kidney; Table S7: Adjusted P Value for glucose-6-phosphate dehydrogenase (G6PDH) test in liver and kidney; Table S8: Adjusted P Value for lipid peroxidation (TBARS) test in liver and kidney; Table S9: Adjusted P Value for thiol groups test in liver and kidney; Table S10: Adjusted P Value for total collagen (pixel/área) test in liver and kidney; Table S11: Adjusted P Value for Number of glomeruli test in kidney.
