# Supplementary material for: Effects of an L-Leucine-Rich Diet on Liver and Kidneys in a Doxorubicin Toxicity Model
Source: Life (Basel). 2023 Aug 29;13(9):1823. doi: 10.3390/life13091823 (PMC10532802; doi:10.3390/life13091823)
Supplement: Supplementary file 1 [file life-13-01823-s001.zip › life-2503203-supplementary.pdf]

Supplementary material

Additional Information from the present study can be found in the table S1 – S11.

Table S1: Liver and kidney function biomarkers

BIOMARKERS	TREATMENT			
	NAIVE	DOXO	LEU	DOX-LEU
ALANINE AMINOTRANSFERASE	25.8±2.7	33.1±2.4	31.6±4.9	33,44±5,54
ASPARTATE AMINOTRANSFERASE	153.5±19.5	150.2±12.8	161.9±9.6	165,22±27,27
CREATININE	0.7±0.03	1.0±0.2	0.8±0.07	0,84±0,2
UREA	40.0±1.7	33.2±1.8	43.0±4.1	36.1±3,52

Table S2: Adjusted P Value for superoxide dismutase (SOD) test in liver and kidney

Tukey's multiple comparisons test	Significant? (p<0.05)	Adjusted P Value
**Liver**		
NAIVE vs. DOX	No	0,9990
NAIVE vs. LEU	No	0,5191
NAIVE vs. DOX+LEU	Yes	0,0425
DOX vs. LEU	No	0,4758
DOX vs. DOX+LEU	Yes	0,0421
LEU vs. DOX+LEU	No	0,4570
**Kidney**		
NAIVE vs. DOX	No	0,9758
NAIVE vs. LEU	No	0,9464
NAIVE vs. DOX+LEU	No	0,9325
DOX vs. LEU	No	0,7741
DOX vs. DOX+LEU	No	0,9978
LEU vs. DOX+LEU	No	0,6693

Table S3: Adjusted P Value for catalase (CAT) test in liver and kidney

Tukey's multiple comparisons test	Significant? (p<0.05)	Adjusted P Value
**Liver**		
NAIVE vs. DOX	No	0,1586
NAIVE vs. LEU	Yes	0,0002
NAIVE vs. DOX+LEU	Yes	<0,0001
DOX vs. LEU	Yes	0,0304
DOX vs. DOX+LEU	Yes	0,0017
LEU vs. DOX+LEU	No	0,5220
**Kidney**		
NAIVE vs. DOX	No	0,1573
NAIVE vs. LEU	No	0,8653
NAIVE vs. DOX+LEU	No	0,2859
DOX vs. LEU	No	0,4313
DOX vs. DOX+LEU	No	0,9769
LEU vs. DOX+LEU	No	0,6647

Table S4: Adjusted P Value for glutathione peroxidase (GPx) test in liver and kidney

**Tukey's multiple comparisons test**	**Significant? (p<0.05)**	**Adjusted P Value**
**Liver**		
NAIVE vs. DOX	No	0,1960
NAIVE vs. LEU	No	0,1931
NAIVE vs. DOX+LEU	No	0,8810
DOX vs. LEU	Yes	0,0034
DOX vs. DOX+LEU	No	0,0532
LEU vs. DOX+LEU	No	0,5442
**Kidney**		
NAIVE vs. DOX	No	0,9923
NAIVE vs. LEU	No	0,9610
NAIVE vs. DOX+LEU	No	0,8676
DOX vs. LEU	No	0,8695
DOX vs. DOX+LEU	No	0,7252
LEU vs. DOX+LEU	No	0,9920

Table S5: Adjusted P Value for reduced glutathione (GSH) test in liver and kidney

**Tukey's multiple comparisons test**	**Significant? (p<0.05)**	**Adjusted P Value**
**Liver**		
NAIVE vs. DOX	Yes	0,0271
NAIVE vs. LEU	No	0,4599
NAIVE vs. DOX+LEU	No	0,9438
DOX vs. LEU	Yes	0,0012
DOX vs. DOX+LEU	Yes	0,0087
LEU vs. DOX+LEU	No	0,7845
**Kidney**		
NAIVE vs. DOX	No	0,8629
NAIVE vs. LEU	No	0,5048
NAIVE vs. DOX+LEU	No	0,9579
DOX vs. LEU	No	0,9051
DOX vs. DOX+LEU	No	0,6086
LEU vs. DOX+LEU	No	0,2892

Table S6: Adjusted P Value for glutathione reductase (GR) test in liver and kidney

**Tukey's multiple comparisons test**	**Significant? (p<0.05)**	**Adjusted P Value**
**Liver**		
NAIVE vs. DOX	Yes	0,0281
NAIVE vs. LEU	No	0,9962
NAIVE vs. DOX+LEU	No	0,7418
DOX vs. LEU	No	0,0560
DOX vs. DOX+LEU	No	0,1779
LEU vs. DOX+LEU	No	0,8751
**Kidney**		
NAIVE vs. DOX	Yes	0,0030
NAIVE vs. LEU	Yes	<0,0001
NAIVE vs. DOX+LEU	Yes	0,0218
DOX vs. LEU	Yes	0,0009

DOX vs. DOX+LEU	No	0,7021
LEU vs. DOX+LEU	Yes	0,0001

Table S7: Adjusted P Value for glucose-6-phosphate dehydrogenase (G6PDH) test in liver and kidney

**Tukey's multiple comparisons test**	**Significant? (p<0.05)**	**Adjusted P Value**
**Liver**		
NAIVE vs. DOX	No	0,4099
NAIVE vs. LEU	No	0,6899
NAIVE vs. DOX+LEU	No	0,9914
DOX vs. LEU	No	0,0577
DOX vs. DOX+LEU	No	0,2738
LEU vs. DOX+LEU	No	0,8501
**Kidney**		
NAIVE vs. DOX	No	0,8357
NAIVE vs. LEU	No	0,7501
NAIVE vs. DOX+LEU	No	0,8114
DOX vs. LEU	No	0,9969
DOX vs. DOX+LEU	No	0,3591
LEU vs. DOX+LEU	No	0,3013

Table S8: Adjusted P Value for lipid peroxidation (TBARS) test in liver and kidney

**Tukey's multiple comparisons test**	**Significant? (p<0.05)**	**Adjusted P Value**
**Liver**		
NAIVE vs. DOX	No	0,9481
NAIVE vs. LEU	No	0,4774
NAIVE vs. DOX+LEU	No	0,6685
DOX vs. LEU	No	0,8969
DOX vs. DOX+LEU	No	0,4751
LEU vs. DOX+LEU	No	0,0878
**Kidney**		
NAIVE vs. DOX	No	>0,9999
NAIVE vs. LEU	No	0,9972
NAIVE vs. DOX+LEU	No	0,7180
DOX vs. LEU	No	0,9975
DOX vs. DOX+LEU	No	0,7464
LEU vs. DOX+LEU	No	0,6307

Table S9: Adjusted P Value for thiol groups test in liver and kidney

**Tukey's multiple comparisons test**	**Significant? (p<0.05)**	**Adjusted P Value**
**Liver**		
NAIVE vs. DOX	No	0,6218
NAIVE vs. LEU	Yes	0,0290
NAIVE vs. DOX+LEU	No	0,5638
DOX vs. LEU	Yes	0,0043
DOX vs. DOX+LEU	No	0,1128
LEU vs. DOX+LEU	No	0,3158

**Kidney**		
NAIVE vs. DOX	No	0,8480
NAIVE vs. LEU	Yes	0,0354
NAIVE vs. DOX+LEU	No	0,0767
DOX vs. LEU	Yes	0,0088
DOX vs. DOX+LEU	Yes	0,0218
LEU vs. DOX+LEU	No	0,9981

Table S10: Adjusted P Value for total collagen (pixel / área) test in liver and kidney

**Tukey's multiple comparisons test**	**Significant? (p<0.05)**	**Adjusted P Value**
**Liver**		
NAIVE vs. DOX	No	0,9999
NAIVE vs. LEU	Yes	0,0012
NAIVE vs. DOX+LEU	No	0,3695
DOX vs. LEU	Yes	0,0014
DOX vs. DOX+LEU	No	0,4001
LEU vs. DOX+LEU	No	0,3466
**Kidney**		
NAIVE vs. DOX	Yes	0,0087
NAIVE vs. LEU	No	0,1764
NAIVE vs. DOX+LEU	No	0,2021
DOX vs. LEU	No	0,5039
DOX vs. DOX+LEU	No	0,5553
LEU vs. DOX+LEU	No	>0,9999

Table S11: Adjusted P Value for Number of glomeruli test in kidney

**Tukey's multiple comparisons test**	**Significant? (p<0.05)**	**Adjusted P Value**
NAIVE vs. DOX	Yes	0,0019
NAIVE vs. LEU	Yes	0,0032
NAIVE vs. DOX+LEU	Yes	0,0029
DOX vs. LEU	No	0,8955
DOX vs. DOX+LEU	No	0,9412
LEU vs. DOX+LEU	No	0,9990
